# The effects of joint hypermobility on pain and functional biomechanics in adolescents with juvenile fibromyalgia: secondary baseline analysis from a pilot randomized controlled trial

**DOI:** 10.1186/s12887-023-04353-y

**Published:** 2023-11-06

**Authors:** William R. Black, Christopher A. DiCesare, Laura A. Wright, Staci Thomas, Megan Pfeiffer, Katie Kitchen, Tracy V. Ting, Sara E. Williams, Gregory D. Myer, Susmita Kashikar-Zuck

**Affiliations:** 1 Department of Pediatrics, University of Kansas School of Medicine, Kansas City, KS USA; 2 Center for Biobehavioral Health, Abigail Wexner Research Institute, Nationwide Children’s Hospital, Columbus, USA; 3 Division of Sports Medicine, Cincinnati Children’s Hospital Medical Center, Cincinnati, OH USA; 4 Department of Neuropsychology, Children’s Healthcare of Atlanta, Atlanta, GA USA; 5 Division of Behavioral Medicine & Clinical Psychology, Cincinnati Children’s Hospital Medical Center, Cincinnati, OH USA; 6 Division of Rheumatology, Cincinnati Children’s Hospital Medical Center, Cincinnati, OH USA; 7 Department of Pediatrics, University of Cincinnati College of Medicine, Cincinnati, OH USA; 8 Emory Sports Performance and Research Center (SPARC), Flowery Branch, GA USA; 9 Emory Sports Medicine Center, Atlanta, GA USA; 10 Department of Orthopaedics, Emory University School of Medicine, Atlanta, GA USA; 11 The Micheli Center for Sports Injury Prevention, Waltham, MA USA

**Keywords:** Chronic pain, Hypermobility, Chronic pain, Biomechanics

## Abstract

**Background:**

Joint hypermobility is a common clinical finding amongst hereditary connective tissue disorders that is observed in pediatric rheumatological settings, and often associated with chronic pain. Joint hypermobility may also contribute to deficits in physical functioning and physical activity, but previous findings have been inconsistent. It is possible that physical activity impairment in joint hypermobility may be due to chronic aberrant movement patterns subsequent to increased joint laxity.

**Method:**

As part of a larger randomized pilot trial of juvenile onset fibromyalgia (JFM), a secondary analysis was conducted to explore whether adolescents with JFM and joint hypermobility differed from non-joint hypermobility peers in terms of pain, daily functioning, and biomechanics (i.e., kinetics and kinematics) during a moderately vigorous functional task.

**Results:**

From the larger sample of adolescents with JFM (*N* = 36), 13 adolescents (36.1%) met criteria for joint hypermobility and 23 did not have joint hypermobility. Those with joint hypermobility exhibited poorer overall functioning (*Md* = 20, *Q*_*1*_*,Q*_*3*_* [5.8, 7.6]* vs. *Md* = 29, *Q*_*1*_*,Q*_*3*_* [5.1, 7.6]*) but there were no differences in pain (*Md* = 6.9, *Q*_*1*_*,Q*_*3*_* [22, 33],* vs. *Md* = 6.45, *Q*_*1*_*,Q*_*3*_* [15, 29.5]*). Inspection of time-series plots suggests those with joint hypermobility exhibited decreased hip flexion and frontal plane hip moment (e.g., resistance to dynamic valgus) during the landing phase (early stance) and greater hip and knee transverse plane moments during the propulsion phase (late stance) of the drop vertical jump task (DVJ). No other differences in lower extremity biomechanics were observed between study groups.

**Conclusions:**

In this exploratory study, there were small but notable differences in biomechanics between patients with JFM who also had joint hypermobility versus those without joint hypermobility during a landing and jumping task (e.g., DVJ). These differences may indicate decreased joint stiffness during landing, associated with increased joint laxity and decreased joint stability, which may put them at greater risk for injury. Further study with a larger sample size is warranted to examine whether these biomechanical differences in patients with JFM and joint hypermobility affect their response to typical physical therapy or exercise recommendations.

## Background

Joint hypermobility, characterized by excessive rangeof movement, is observed in 7–36% of children and adolescents [1]. Joint hypermobility is also a primary clinical finding among individuals with hereditary connective tissue disorders (e.g., Ehlers-Danlos Syndromes [EDS], Hypermobility Spectrum Disorder [previously known as Joint Hypermobility Syndrome]), and is frequently observed in youth (40%) diagnosed with idiopathic chronic musculoskeletal pain musculoskeletal pain conditions, such as juvenile onset fibromyalgia [JFM] [2, 3]. Joint hypermobility and associated musculoskeletal pain are primary clinical features frequently referred to pediatric rheumatology settings, even in the absence of identified rheumatologic disease [4, 5]. As many as 40% of adolescents with JFM also exhibit joint hypermobility; however, research on joint hypermobility and chronic musculoskeletal pain is mixed.

Joint hypermobility does not appear to be directly linked to physical activity impairment but is associated with repetitive use injuries [6, 7], and may be indirectly associated with physical impairment through altered compensatory biomechanics due to increased joint laxity [8]; this alteration in biomechanics may then lead to a higher risk for injury and pain [9, 10]. Various abnormalities in gait characteristics (i.e., toe-walking, abnormal gait patterns, delayed walking) [11, 12]; and knee motion (i.e., higher knee extension and flexion) [13] are present in youth with joint hypermobility. Furthermore, these youth exhibit sensorimotor deficits in knee joint proprioception, critical for controlling balance and knee extensor and flexor muscle torque [14]. The association of potential biomechanical and movement differences with measures of self-reported deficits in physical functioning remains unclear.

Joint hypermobility may also be a risk factor for the development of widespread musculoskeletal pain during later adolescence, such as exhibited in JFM [15, 16]. Ting et al., (2012) found that in a sample of adolescents with JFM, joint hypermobility patients demonstrated higher sensitivity to mechanical pain (i.e., lower tender point thresholds) and reported a greater number of painful tender points [3]. Additionally, children with joint hypermobility exhibit substantially reduced maximal exercise capacity compared to age- and gender-matched controls [17]. However, it is unclear whether joint hypermobility is associated with deficits in physical functioning. Leone et al. (2009) found hypermobility was associated with less disability in daily activities and increased physical activity [18]. In other work, joint hypermobility was unrelated to both self-reported physical activity (e.g., daily metabolic equivalents in school, sports, leisure time) and pain [19]. Furthermore, adolescent athletes with joint hypermobility report better overall functioning and pain than those with joint hypermobility who do not engage in sports [20, 21].

The aim of this study was to better understand how joint hypermobility may affect functioning in a sample of adolescents with JFM, given the high degree of clinical overlap. We performed a secondary analysis of data collected as part of a pilot randomized clinical trial [22] to explore whether adolescents with JFM and joint hypermobility differed from non-joint hypermobility peers in terms of pain, daily functioning, and biomechanics. Given the added mechanical stress of joint laxity, it was hypothesized that youth with chronic pain and joint hypermobility would demonstrate higher levels of pain intensity and greater functional deficits across landing biomechanics compared to those with chronic pain but without joint hypermobility. This study has the potential to advance our understanding of potential sub-groups of patients which widespread musculoskeletal pain, such as those with joint hypermobility, and potential clinical implications of this co-occurring condition.

## Findings

### Methods

#### Participants

Adolescents (between 12 and 18 years of age), that met criteria for JFM, and had at least moderate functional disability and pain, were recruited as part of a larger pilot randomized clinical trial for teens with JFM, which tested a combined cognitive-behavioral therapy and neuromuscular exercise training program; only baseline data were included in this study [22, 23]. Thirty-six female adolescents (*M*_age_ = 15.61, *SD* = 1.42) participated in the study; while both males and females were eligible, 90% of the overall study sample were female, and only females had valid biomechanics data. This study was approved by the Institutional Review Board of the mid-western pediatric hospital where the study was carried out and the parent trial was registered on clinicaltrials.gov (NCT #R21AR063412). Enrollment occurred from December 16, 2013 to April 1, 2016 and follow-up occurred from July 28, 2014 to August 31, 2016.

#### Measures

##### Hypermobility

The Beighton Score [24] was used to assess generalized joint hypermobility. The Beighton Score system has a total of 9 points with one point allotted to each hypermobile joint (lower back and bilateral elbows, knees, thumbs, and 5^th^digits). Cutpoints to define joint hypermobility in pediatrics range from 5–6 [25, 26]. For the purposes of this study, we used a Beighton score of ≥ 5 as an indicator of joint hypermobility because our sample is an adolescent sample/range, and this cutoff has been recommended previously in females older than 8 years of age [25–27]. Additionally, lower-limb joint hypermobility is described as the sum of scores for the left knee, right knee, and hips (i.e., palms on the floor), and is captured as the total score out of 3 [25].

##### Pain intensity

Participants rated their average pain intensity over the past 2 weeks using a 0–10 cm Visual Analog Scale (VAS) ranging from 0 (no pain) to 10 (worst possible pain). The VAS has been well-validated among youth with chronic pain [28].

##### Functional disability

The Functional Disability Inventory (FDI) is a 15-item, 5-point Likert scale (0 – no trouble; 4 – impossible) that assesses adolescents’ perceived difficulty with daily activities due to their physical health (e.g., “Doing chores at home”) [29]. Adolescents rated their perception of activity limitations over the last few days, with higher scores indicative of greater disability.

##### Functional biomechanical assessment

**Fig. 1:**
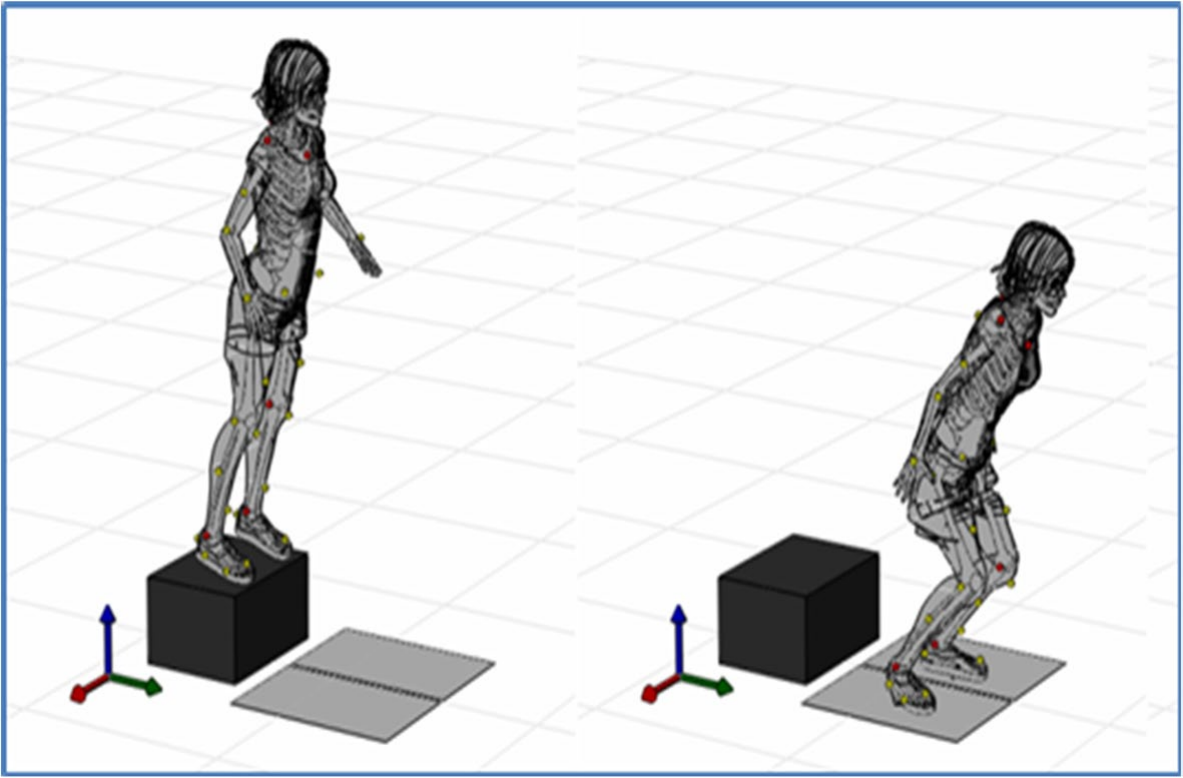
Drop vertical jump task

The methodology for biomechanical assessment, data processing, andanalyses used in the pilot randomized trial are fully described in prior publications [30, 31]. Briefly, we used 3-D motion capture of participants performing a standard DVJ task to examine knee and hip kinetics and kinematics (see Fig. 1) [30].

### Data analyses

**Fig. 2:**
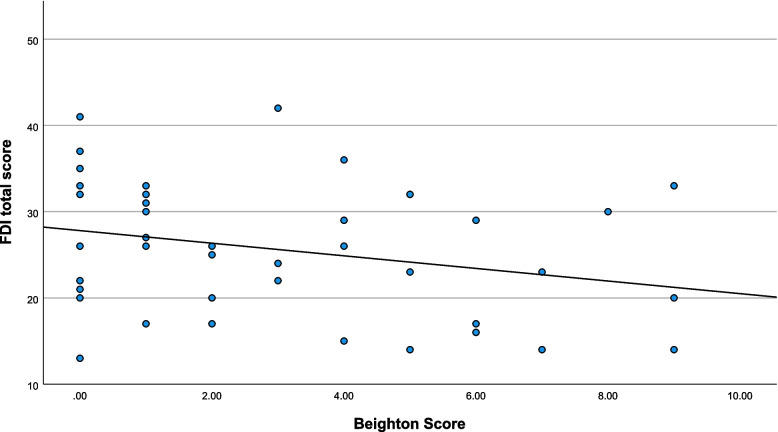
Scatterplot of Beighton and FDI scores

**Fig. 3:**
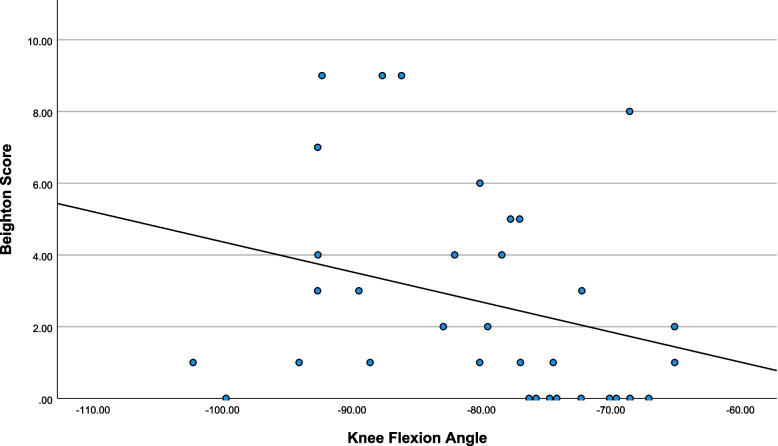
Scatterplot of Beighton and knee flexion angle

**Fig. 4:**
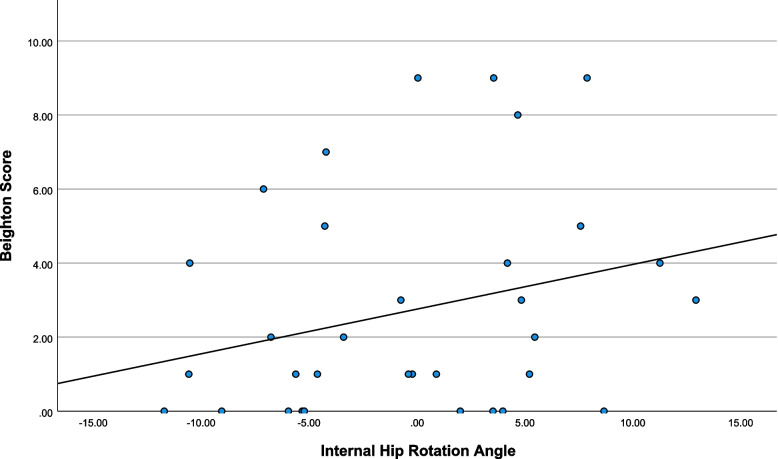
Scatterplot of Beighton and internal hip rotation angle

**Fig. 5:**
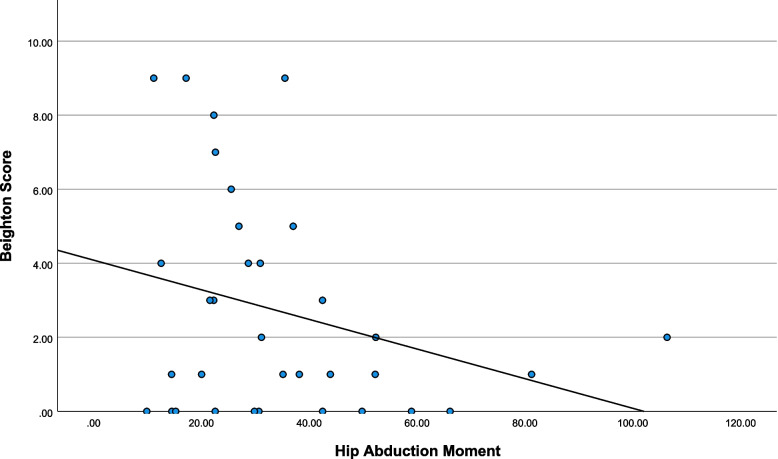
Scatterplot of Beighton and hip abductor moment

**Fig. 6:**
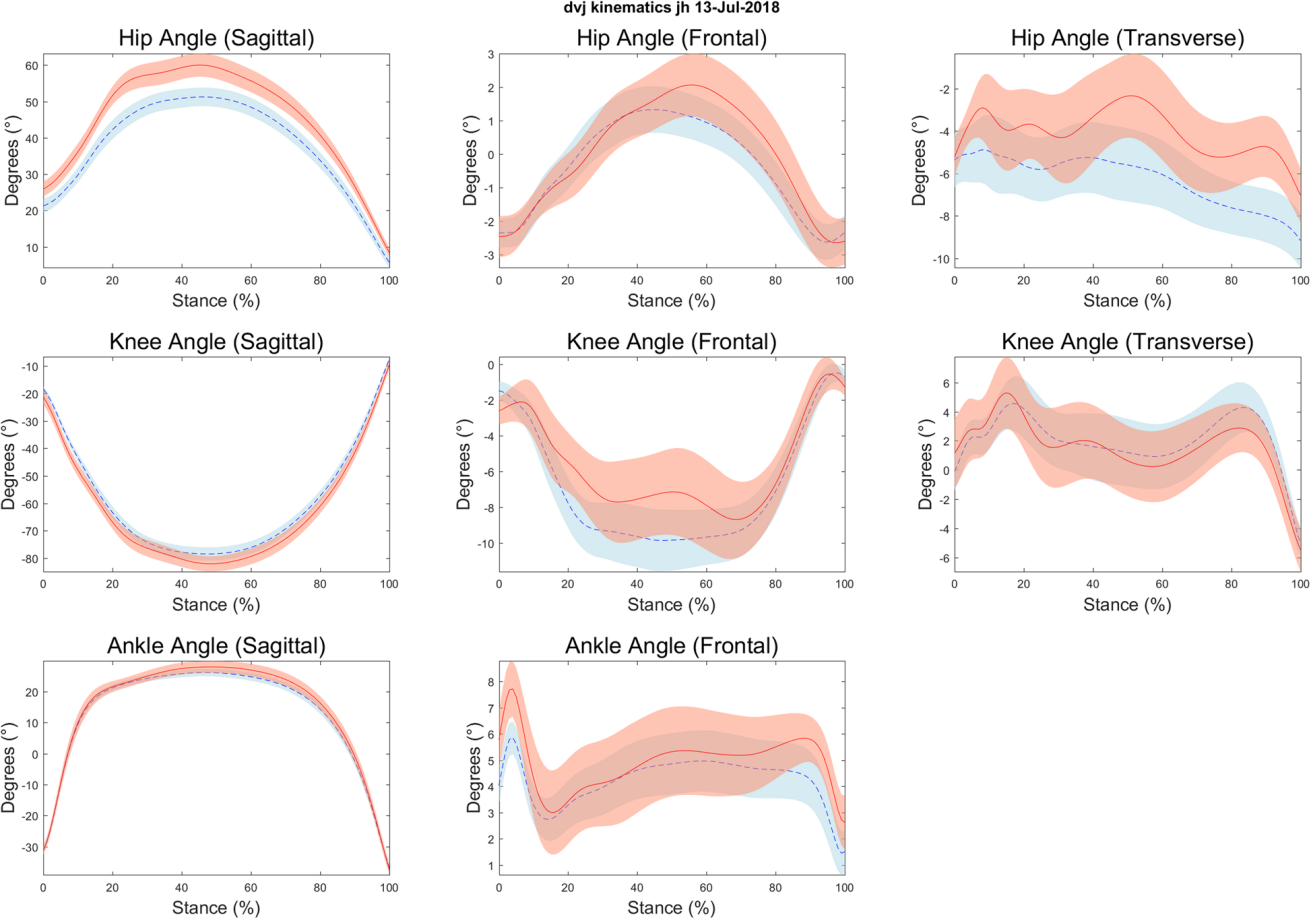
Kinematic time-series plots for joint hypermobility vs. non-hypermobility during drop vertical jump task

**Fig. 7:**
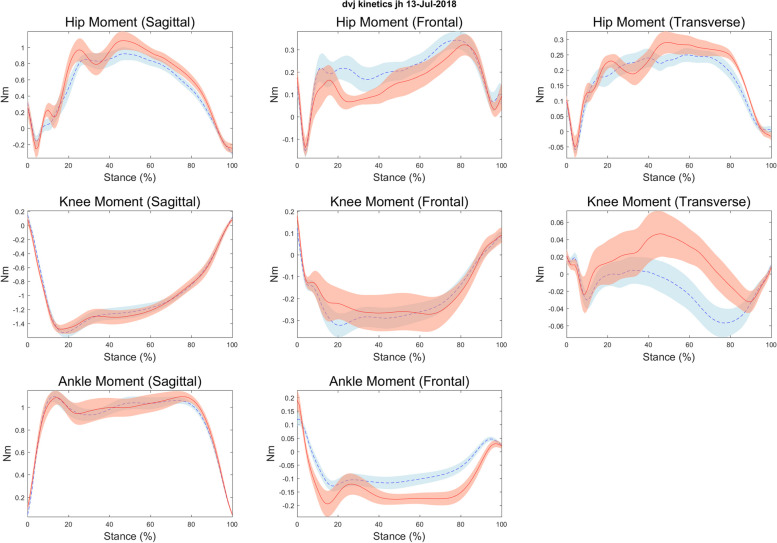
Kinetic time-series plots for joint hypermobility vs. non-hypermobility during drop vertical jump task

Descriptive statistics are presented in Table 1 and scatterplots of notable relationships are presented in Figs. 2, 3, 4 and 5. Kinematic (Fig. 6) and kinetic (Fig. 7) time-series plots for the mean values across the stance phase of the DVJ with shaded areas of standard error are each presented for joint hypermobility and non-joint hypermobility groups. Non-overlapping areas of the standard error (i.e., gaps or white areas in between the shaded group-based confidence intervals) constitute significant differences in biomechanics across the DVJ.

**Table 1:** Descriptive statistics among study variables – non-hypermobile vs. hypermobile

	**Total Sample (*n* = 36)**			**Non-JH (*n* = 23)**				**JH (*n* = 13)**			
Variable	*M* (*SD*)	Md	CI (95%)	*M* (*SD*)	Md	CI (95%)	*Q1,Q3*	*M* (*SD*)	Md	CI (95%)	*Q1,Q3*
Pain intensity	6.56 (1.40)	6.85	6.06, 7.06	6.70 (1.40)	6.90	6.02, 7.34	5.8, 7.6	6.30 (1.15)	6.45	5.78, 7.12	5.1, 7.6
FDI	26.36 (7.63)	26	23.4, 29.43	28.79 (7.33)	27.0	25.26, 32.32	22, 33	21.90 (7.31)	20.00	16.66, 27.14	15, 29.5
Beighton score	3.33 (2.96)	3	2.18, 4.51	1.47 (1.50)	1.00	0.86, 2.20	1, 4	6.90 (1.72)	6.50	5.66, 8.14	6, 9
Lower limb Beighton	1.16 (1.18)	1	0.78, 1.55	0.52 (0.15)	0.00	0.22, 0.82	0, 1	2.31 (0.95)	3.00	1.79, 2.82	2, 3

## Results

### Descriptive statistics

Of the 36 patients enrolled in the larger study [22, 23], 13 (36.1%) met criteria for joint hypermobility based on a Beighton score ≥ 5. Participants demonstrated moderately elevated levels of functional disability (*M* = 26.36) and pain intensity (*M* = 6.56). Descriptive statistics between the hypermobility and non-hypermobility groups are presented in Table 1.

### Relations between hypermobility, and biomechanics, pain, and functional disability

Beighton scores decreased as functional disability increased (Fig. 2), indicating that increased Beighton scores are associated with lower disability. Higher Beighton scores may also be associated with higher flexion (Fig. 3). Increased Beighton scores may also trend towards increased peak hip internal rotation angle (Fig. 4) and decreased hip abductor moment (Fig. 5).

### Hypermobility group comparisons

Functional disability was lower for those with joint hypermobility (*Md* = 20) compared to those without joint hypermobility (*Md* = 27), thus, participants who met clinical criteria for joint hypermobility reported significantly less physical impairment. Pain intensity was similar between joint hypermobility (*Md* = 6.45) and non-joint hypermobility (*Md* = 6.90) groups.

#### Time-series assessment of function

Qualitative visual evaluation of the kinematic and kinetic time-series plots showed some differences between joint hypermobility and non-joint hypermobility participants. The joint hypermobility group demonstrated greater hip flexion than the non-joint hypermobility group throughout the entire DVJ (Fig. 6). No other differences in hip or knee kinematics were observed. Kinetic plots demonstrated similar relationships, with two exceptions. While kinematics during the DVJ were similar between the non-joint hypermobility and joint hypermobility groups, those with joint hypermobility exhibited decreased hip frontal plane hip abduction moment during the landing phase early and greater hip and knee transverse plane moment during late take off phase, denoted by non-overlapping error bars, as seen in Fig. 7.

## Discussion

The results of this pilot study suggest that adolescents with JFM and joint hypermobility show similar clinical pain intensity levels to those with JFM without joint hypermobility; however, those with joint hypermobility had lower levels of overall physical impairment. We found small differences in biomechanics between joint hypermobility groups during a landing and jumping task (e.g., hip [sagittal] angle; hip [frontal and transverse] and knee [transverse] moments). Specifically, those with joint hypermobility may demonstrate greater hip flexion (Fig. 6), decreased hip abduction moment (Fig. 7), and potentially greater knee flexion compared to those without joint hypermobility.

Our preliminary findings indicate among adolescents with JFM, those with joint hypermobility may demonstrate decreased joint stiffness during landing, a biomechanical feature that is consistent with excessive joint laxity [32]. Study findings also suggest other differences in movement among those with joint hypermobility, including greater transverse plane hip and knee moment during propulsion (near the end of the stance phase; Fig. 7), that were not observed in those without joint hypermobility. Taken together, these findings could indicate that JFM patients with joint hypermobility may demonstrate differences in hip position and movement compared to those without joint hypermobility. Such differences could be associated with increased forces exerted in the knees, which has been associated with increased risk for injury in adolescent athletes [33]. Joint stiffness or greater muscular supported movement has been shown to be protective against lower extremity injury risk [34]; conversely, reduced stiffness, as potentially demonstrated in this task among individuals with joint hypermobility, would indicate differences in how they compensate when landing compared to individuals without joint hypermobility which could pose a greater risk for injury. These pilot results are consistent with previous research demonstrating that individuals with joint hypermobility may be prone to differences in knee biomechanics [12, 13]. Further study is warranted to examine whether these biomechanical differences in joint hypermobility patients affect how they respond to typical physical therapy or exercise recommendations.

This pilot study has several limitations. First, the small sample size may not have provided enough power to detect significant group differences. Additionally, this study was a secondary analysis involving participants with a primary diagnosis of JFM, and results may not generalize to other chronic musculoskeletal pain conditions or those with joint hypermobility without chronic pain at all. In addition, joint hypermobility group classifications were based on adolescents’ Beighton scores, and there is some debate over appropriate clinical cut-off scores to use in pediatric populations [35]. Beighton scores also do not capture joint laxity in other prominent areas of the body that were pertinent to strength assessments in the current study (e.g., hips, internal/external rotation of knees).

## Conclusion

These results tentatively support the use of neuromuscular training and exercise programming in adolescents with chronic musculoskeletal pain, regardless of joint hypermobility status; though, additional work is needed to address concerns regarding our study findings being attributed to error alone. However, findings also do suggest subtle differences in biomechanics in adolescents with JFM who also have joint hypermobility. Based on these preliminary findings, these differences in body biomechanics may be worth examining in more definitive studies with larger samples, to determine how exercise programs can be best modified for patients with joint hypermobility.

## Data Availability

Given the size and complexity of the biomechanics files, data may be provided and shared upon a reasonable request.

## Abbreviations

EDS: Ehlers-Danlos Syndromes
joint hypermobility: Joint Hypermobility
HSD: Hypermobility Spectrum Disorder
musculoskeletal pain: Musculoskeletal Pain
JFM: Juvenile Fibromyalgia
VAS: Visual Analog Scale
FDI: Functional Disability Inventory
DVJ: Drop Vertical Jump

## References

[1] Scheper MC, Engelbert RH, Rameckers EA, Verbunt J, Remvig L, Juul-Kristensen B (2013). Children with generalised joint hypermobility and musculoskeletal complaints: state of the art on diagnostics, clinical characteristics, and treatment. Biomed Res Int.

[2] Gedalia A, Press J, Klein M, Buskila D (1993). Joint hypermobility and fibromyalgia in schoolchildren. Ann Rheum Dis.

[3] Ting TV, Hashkes PJ, Schikler K, Desai AM, Spalding S, Kashikar-Zuck S (2012). The role of benign joint hypermobility in the pain experience in Juvenile Fibromyalgia: an observational study. Pediatr Rheumatol.

[4] Hakim A, Grahame R (2003). Joint hypermobility. Best Pract Res Clin Rheumatol.

[5] Kerr A, Macmillan C, Uttley W, Luqmani R (2000). Physiotherapy for children with hypermobility syndrome. Physiotherapy.

[6] Hudson N, Fitzcharles MA, Cohen M, Starr MR, Esdaile JM (1998). The association of soft-tissue rheumatism and hypermobility. Br J Rheumatol.

[7] Cowderoy GA, Lisle DA, O'Connell PT (2009). Overuse and impingement syndromes of the shoulder in the athlete. Magn Reson Imaging Clin N Am.

[8] Tinkle BT, Levy HP (2019). Symptomatic joint hypermobility: the hypermobile type of Ehlers-Danlos syndrome and the hypermobility spectrum disorders. Med Clin North Am.

[9] Svoboda Z, Honzikova L, Janura M, Vidal T, Martinaskova E (2014). Kinematic gait analysis in children with valgus deformity of the hindfoot. Acta Bioeng Biomech.

[10] Kothari A, Dixon PC, Stebbins J, Zavatsky AB, Theologis T (2016). Are flexible flat feet associated with proximal joint problems in children?. Gait Posture.

[11] Adib N, Davies K, Grahame R, Woo P, Murray KJ (2005). Joint hypermobility syndrome in childhood. A not so benign multisystem disorder?. Rheumatology (Oxford).

[12] Engelbert RH, Uiterwaal CS, Gerver WJ, van der Net JJ, Pruijs HE, Helders PJ (2004). Osteogenesis imperfecta in childhood: impairment and disability. A prospective study with 4-year follow-up. Arch Phys Med Rehabil..

[13] Fatoye FA, Palmer S, van der Linden ML, Rowe PJ, Macmillan F (2011). Gait kinematics and passive knee joint range of motion in children with hypermobility syndrome. Gait Posture.

[14] Fatoye F, Palmer S, Macmillan F, Rowe P, van der Linden M (2009). Proprioception and muscle torque deficits in children with hypermobility syndrome. Rheumatology (Oxford).

[15] Tobias JH, Deere K, Palmer S, Clark EM, Clinch J (2013). Joint hypermobility is a risk factor for musculoskeletal pain during adolescence: findings of a prospective cohort study. Arthritis Rheum.

[16] Sohrbeck-Nøhr O, Kristensen JH, Boyle E, Remvig L, Juul-Kristensen B (2014). Generalized joint hypermobility in childhood is a possible risk for the development of joint pain in adolescence: a cohort study. BMC Pediatr.

[17] Engelbert RH, van Bergen M, Henneken T, Helders PJ, Takken T (2006). Exercise tolerance in children and adolescents with musculoskeletal pain in joint hypermobility and joint hypomobility syndrome. Pediatrics.

[18] Leone V, Tornese G, Zerial M (2009). Joint hypermobility and its relationship to musculoskeletal pain in schoolchildren: a cross-sectional study. Arch Dis Child.

[19] Juul-Kristensen B, Kristensen JH, Frausing B, Jensen DV, Rogind H, Remvig L (2009). Motor competence and physical activity in 8-year-old school children with generalized joint hypermobility. Pediatrics.

[20] Nicholson LL, Adams RD, Tofts L, Pacey V (2017). Physical and psychosocial characteristics of current child dancers and nondancers with systemic joint hypermobility: a descriptive analysis. J Orthop Sports Phys Ther.

[21] Schmidt H, Pedersen TL, Junge T, Engelbert R, Juul-Kristensen B (2017). Hypermobility in adolescent athletes: pain, functional ability, quality of life, and musculoskeletal injuries. J Orthop Sports Phys Ther.

[22] Kashikar-Zuck S, Black WR, Pfeiffer M, et al. Pilot Randomized Trial of Integrated Cognitive-Behavioral Therapy and Neuromuscular Training for Juvenile Fibromyalgia: The FIT Teens Program. J Pain. 2018. 10.1016/j.jpain.2018.04.003. 10.1016/j.jpain.2018.04.003 PMC6119635 29678563

[23] Black WR, DiCesare CA, Thomas S (2021). Preliminary Evidence for the Fibromyalgia Integrative Training Program (FIT Teens) Improving Strength and Movement Biomechanics in Juvenile Fibromyalgia: Secondary Analysis and Results from a Pilot Randomized Clinical Trial. Clin J Pain.

[24] Beighton P, Horan F (1969). Orthopaedic aspects of the Ehlers-Danlos syndrome. J Bone Joint Surg Br.

[25] Nicholson LL, Chan C, Tofts L, Pacey V (2022). Hypermobility syndromes in children and adolescents: Assessment, diagnosis and multidisciplinary management. Aust J Gen Pract.

[26] Singh H, McKay M, Baldwin J (2017). Beighton scores and cut-offs across the lifespan: cross-sectional study of an Australian population. Rheumatology.

[27] Nicholson LL, Simmonds J, Pacey V (2022). International perspectives on joint hypermobility: a synthesis of current science to guide clinical and research directions. JCR.

[28] McGrath PJ, Walco GA, Turk DC (2008). Core outcome domains and measures for pediatric acute and chronic/recurrent pain clinical trials: PedIMMPACT recommendations. J Pain.

[29] Walker LS, Greene JW (1991). The functional disability inventory: measuring a neglected dimension of child health status. J Pediatr Psychol.

[30] Sil S, Thomas S, DiCesare C (2015). Preliminary evidence of altered biomechanics in adolescents with juvenile fibromyalgia. Arthritis Care Res.

[31] Tran ST, Thomas S, DiCesare C (2016). A pilot study of biomechanical assessment before and after an integrative training program for adolescents with juvenile fibromyalgia. Pediatr Rheumatol.

[32] Shultz SJ, Pye ML, Montgomery MM, Schmitz RJ (2012). Associations between lower extremity muscle mass and multiplanar knee laxity and stiffness: a potential explanation for sex differences in frontal and transverse plane knee laxity. Am J Sports Med.

[33] Myer G, Brent J, Ford K, Hewett T (2008). A pilot study to determine the effect of trunk and hip focused neuromuscular training on hip and knee isokinetic strength. Br J Sports Med.

[34] Ford KR, Myer GD, Hewett TE (2010). Longitudinal effects of maturation on lower extremity joint stiffness in adolescent athletes. Am J Sports Med.

[35] Juul-Kristensen B, Schmedling K, Rombaut L, Lund H, Engelbert RH (2017). Measurement properties of clinical assessment methods for classifying generalized joint hypermobility-A systematic review. Am J Med Genet C Semin Med Genet.

